# Avian leukosis virus subgroup J associated with the outbreak of erythroblastosis in chickens in China

**DOI:** 10.1186/1743-422X-10-92

**Published:** 2013-03-22

**Authors:** Guihua Wang, Yanping Jiang, Linin Yu, Yue Wang, Xiaomin Zhao, Ziqiang Cheng

**Affiliations:** 1 Department of Fundamental Veterinary, Molecular pathology lab, College of Veterinary Medicine, Shandong Agricultural University, Tai’an, China; 2 Shandong Provincial Key Laboratory of Animal Biotechnology and Disease Control and Prevention, Shandong Agricultural University, Tai’an, China

**Keywords:** Erythroblastosis, Avian leukosis virus subgroup J, PCR, Immunohistochemistry

## Abstract

**Background:**

Emaciation, depression and lethargy were observed in two flocks of Chinese local breed and one flock of commercial layer chicken infected naturally from 2010 to 2011. The aims of this study were to diagnose.

**Methods and results:**

Gross observation showed that severe enlargement of liver, spleen and kidney, and hemorrhage of thymus, muscle and glandular stomach in all submitted birds. The liver and lung of one flock had diffuse, multifocal white raised foci on the surface as well as on the cut-surface. Numerous erythrocytoblasts with bigger volume, basophilic cytoplasm and round nucleus were observed in blood and bone marrow smears. The same erythrocytoblasts were also found crowded in blood vessels and mesenchym of tissues by histological examination, and some had mitotic figures. PCR results showed that three flocks were positive for ALV-J with specific fragment of 924 bp, negative for AEV, ALV-A, ALV-B, Marek’s disease virus (MDV) and Reticuloendotheliosis virus (REV). The results of immunohistochemistry showed that cytoplasm of histiocytes and erythrocytoblasts in lung and spleen sections was positive for ALV-J antigen.

**Conclusion:**

These data demonstrated that erythroblastosis was all induced by ALV-J in the three different flocks. This is the first document report of erythroblastosis induced by ALV-J in China flocks.

## Background

Avian leukosis virus subgroup J (ALV-J) is an oncogenic exogenous retrovirus first isolated in the late 1980s and reported in 1991 [1]. The hosts with clinical infection of ALV-J are characterized as immune tolerance, high mortality, delayed growth, and development of a variety of tumors including myelocytomas, sarcomas, hemangiomas, nephromas and erythroblastosis [2-5]. The erythroblastosis is a neoplastic disease induced by viral disoperation for erythroblast in bone marrow. In 1988, Houghton et al. found that the chicken erythroblastosis was associated with ALV-J during their investigation of the neoplastic disease of broilers with experimental infections of ALV-J [6]. Venugopal *et al.* (2000) observed the indicative lesions of erythroblastosis in tissues from flocks with suspicion of ALV-J infection. However, the chicken erythroblastosis induced by ALV-J has never been identified in China.

In the present study, we identified the chicken erythroblastosis that was associated with natural infections of ALV-J in two flocks of Chinese local breed and one flock of commercial layer chicken from 2010 to 2011. This is the first report of the chicken erythroblastosis induced by ALV-J in China.

## Materials and methods

### Ethical approval

This study was carried out in strict adherence to the recommendations in the Guide for the Care and Use of Laboratory Animals of the National Institutes of Healthy. The protocol was approved by the Committee on the Ethics of Animal of Shandong (Permit Number: 20100326).

### Case history

From the year 2010 to 2011, our laboratory (The Molecular Pathology Laboratory, College of Veterinary Medicine, Shandong Agricultural University) received six sick representatives of the 20-day-old commercial layer chickens (flock 1), three 90-day-old (flock 2) and three 110-day-old (flock 3) Chinese local breed chickens for the diagnostic purpose. The birds of flock 1 presented depression, recumbency and pale cockscomb started from 15 days of age, and the mortality in the population was 18%. The birds of flock 2 showed symptoms of nerve system disorders such as depression and ataxia started from 85-day-old, and the mortality was 12%. The birds of flock 3 anorexia, lethargy and emaciation started from 90-day-old, and the mortality reached 20% at 100-day-old.

### Histopathological examination

The samples of the liver, spleen, kidney, heart, lung, proventriculus, sciatic nerve, brain, and bone marrow were collected and fixed in 10% buffered neutral formalin. The fixed tissues were embedded in paraffin, sectioned at 4 μm thick, and stained with haematoxylin and eosin. The sample slides were observed under light microscopy.

### Polymerase chain reaction (PCR)

DF-1 cells were seeded in 6-well plate at a density of approximately 1×10^6^ cells per well. Tissue extracts from ill chickens were inoculated onto DF-1 and incubated at 37°C for 2 h. Then the cells were cultured with fresh medium contained 1% fetal bovine serum (FBS, Invitrogen, CA, USA). Observed daily, on the seven days of the post-inoculation, provirus DNA were extracted from infected DF-1 cells using DNA extraction kit (TaKaRa, Bio, Inc., Beijing, China). The PCR amplifications using provirus DNA as templates with the primers (Table 1) specific for the avian erythroblastosis virus (AEV) specific primers (Genbank number : K02006.1), ALV-A, ALV-B [7], ALV-J [8], REV [9] and MDV [10] respectively were performed. The amplification of the target gene was set up in a 25 μL reaction containing 1 μL of DNA, 2.5 μL of 10×Taq buffer (TaKaRa, Bio, Inc., Beijing, China), 2.5 μL of dNTP (2.5 mmol/ L), 1 μL of each primer (10 mmol/ L), and 17 μL of ddH_2_O. The PCR products were detected by 0.8% agarose gel electrophoresis with ErBr staining.

**Table 1:** Primers for differential diagnosis

**Primers**	**Sequences**	**Fragment sizes**
AEV(env)	F :5^′^-AGAAGAACCTGCACCCCACCTAC-3^′^	1981bp
	R :5^′^- AAAGACCGATGCCTAGACCAACC-3^′^	
ALV-J(env)	F :5^′^-ATGGGAGTTCATCTATTGCAACAACCAG-3^′^	924bp
	R :5^′^-TTAGCGCCTGCTACGGTGGTGACC-3	
ALV-A(env)	F :5^′^ –CGAGAGTGGCTCGCGAGATGG-3^′^	1300bp
	R :5^′^-CCCATTTGCCTCCTCTCCTTGTA-3^′^	
ALV-B(env)	F :5^′^-CGAGAGTGGCTCGCGAGATGG-3^′^	1100bp
	R :5^′^-AGCCGGACTATCGTATGGGGTAA-3^′^	
MDV(132bp)	F:5^′^-TACTTCCTATATAGATTGAGACGT-3^′^	132bp
	R:5^′^-GAGATCCTCGTAAGGTGTAATATA-3^′^	
REV(LTR)	F:5^′^-CATACTGGAGCCAATGGTT-3^′^	300bp
	R:5^′^ AATGTTGTAGCGAAGTACT-3^′^	

### Immunohistochemistry

To detect the presence of ALV-J and AEV antigen, tissues were fixed with 10% buffered neutral formalin, paraffin-embedded, sectioned with the thickness of 4 μm, and mounted on poly-l-lysine-coated slides. The tissue sections were stained with a routine streptavidin biotin/horseradish peroxidase (HRP)-conjugated immunohistochemical technique as described by [11]. Briefly, the sections were pre-treated with 3% hydrogen peroxide in methanol, and blocked with 5% bovine serum albumin in PBS for 10 min. Then the slides were incubated with primary antibody (a rabbit anti-ALV-J and anti-AEV surface protein prepared by our lab) at a dilution of 1: 400 for 1 h, washed three times with PBS, and incubated with the secondary antibody (biotinylated goat anti-rabbit IgG, Santa Cruz, CA, USA) at a dilution of 1:5000 for 30 min. After three washes, the tertiary conjugate streptavidin/HRP was applied for 30 min. Chromogen (AEC) was applied and developed microscopically for positive straining. The reaction was stopped by water and the slides were then counterstained with hematoxylin. Finally, the slides examined microscopically with light microscopy. In negative immunostaining controls, the primary antibody was replaced with non-immune rabbit IgG.

## Results

### Gross lesions

**Figure 1:**
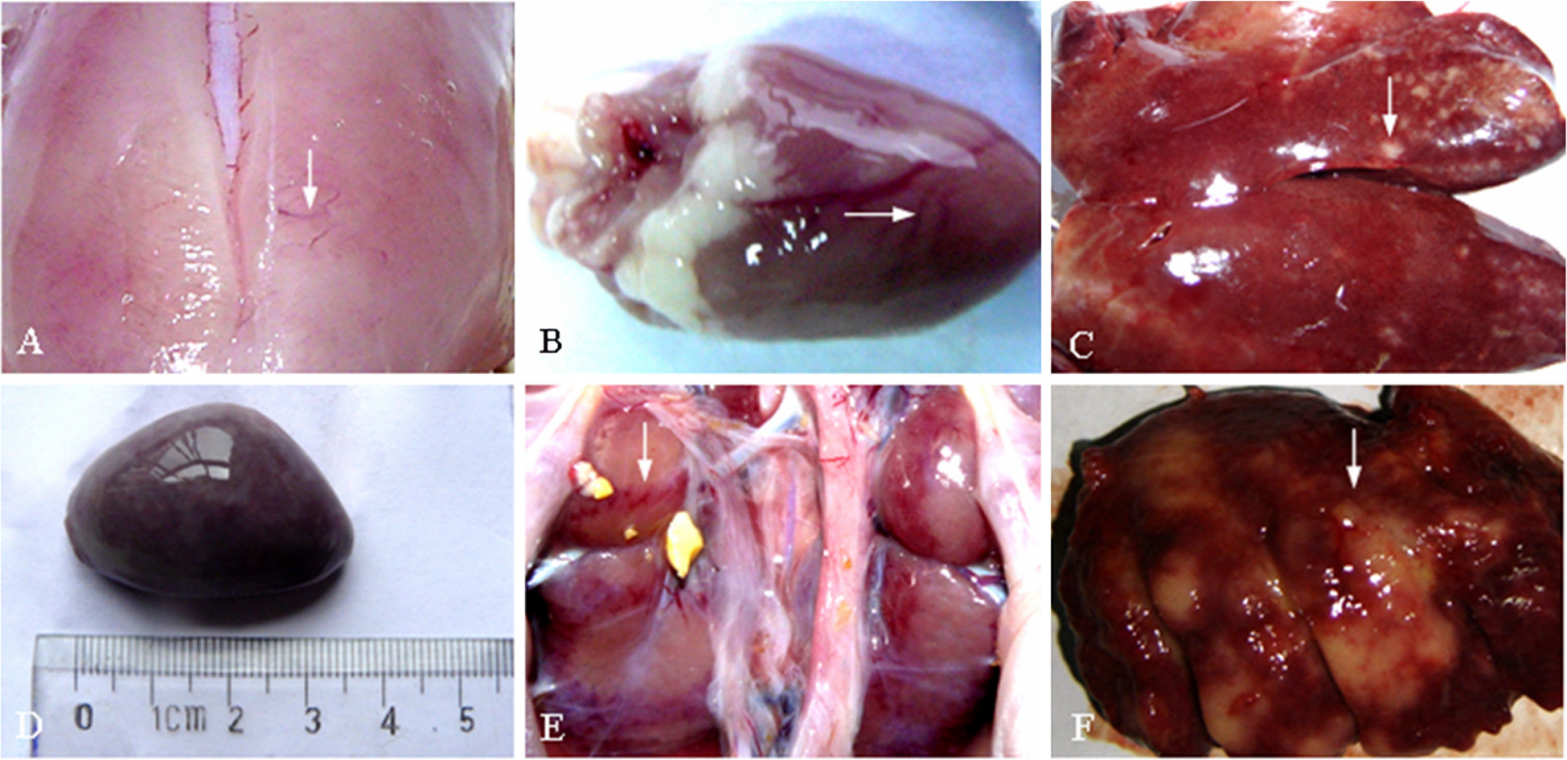
**Gross lesion of submitted birds.** (**A**) hemorrhage on pale pectoral muscle; (**B**) hemorrhage on myocardium; (**C**) liver enlarged with multifocal, white raised foci throughout; (**D**) spleen enlarged; (**E**) kidney with piebald; (**F**) diffuse, multifocal white raised foci on the surface lung.

The birds examined were characterized with pale pectoral muscles, myocardium hemorrhage (Figure 1A-B), and the enlarged visceral organs especial liver with multifocal, white raised foci throughout (Figure 1C), spleen (Figure 1D) and kidney with piebald (Figure 1E) which were brittle fragile. The bone marrow became jelly like with lighter colour. The diffuse, multifocal white raised foci were observed on the lung surface (Figure 1F).

### Histopathology

**Figure 2:**
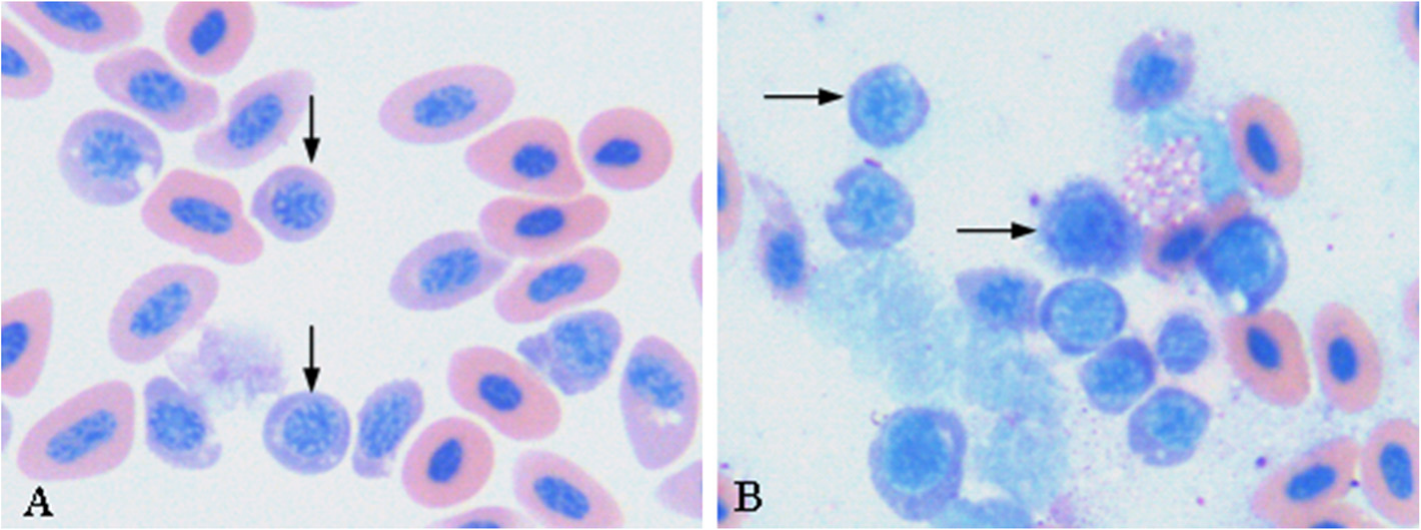
**Blood and bone marrow smears.** (**A**) There was massive replacement of the normal of erythrocyte in blood smears by different periods of erythroblasts in blood smear, 400×; (**B**) The number of erythroblast was severely increased in bone marrow smears, 400×; Giemsa staining.

There were numerous erythroblasts at different growth stages replaced the normal erythrocytes in the blood smears as shown in Figure 2A. The erythroblasts had polymorphism (spherical, ellipse and irregular shape), greater cellularity, loosen chromatin than normal erythrocytes. Their cytoplasm was basophilic and contained vacuolus surrounded the spherical or ellipse nuclei. In the bone marrow smears, the amount of erythroblasts was significantly increased. The erythroblasts had bigger volume, round shape and irregular edge. The features of the cytoplasm, nuclei and chromatin of the bone marrow erythroblasts were similar to that of the blood erythroblasts (Figure 2B).

**Figure 3:**
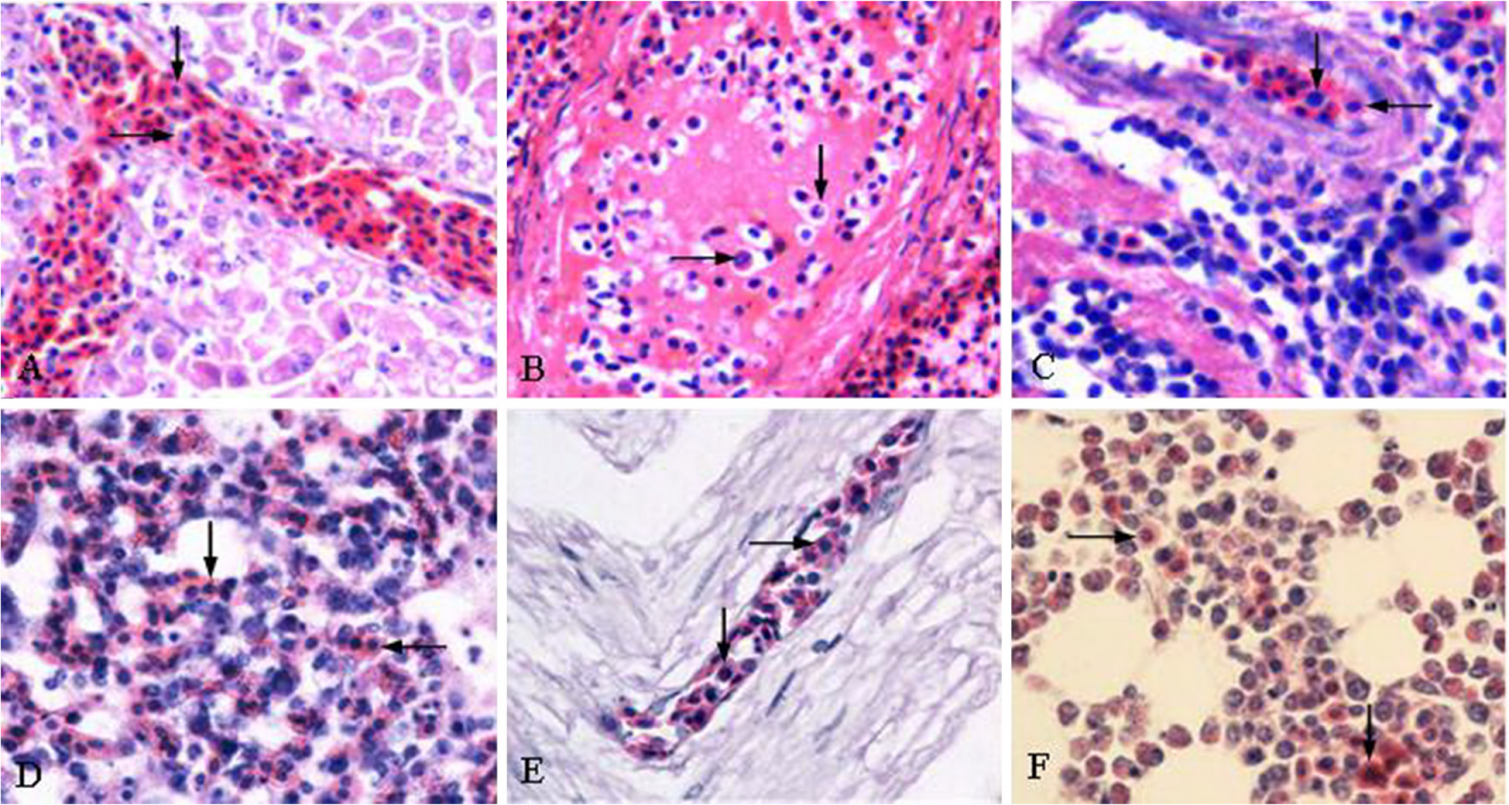
**Histopathology.** Erythroblasts was accumulated in dilated Disse’s space of livers (**A**) and blood capillary in spleen (**B**), myocardium (**C**), lung (**D**) and nerves (**E**), HE, 200×; (**F**) The parenchyma proliferation and increased erythroblast were seen in bone marrow, HE, 200×.

There were some similar pathological changes in various tissues observed under light microscope. Severe hemorrhage and congestion were found in all tissue sections of the examined birds and the normal architecture of all tissues were damaged with different degrees. The Disse’s space of livers (Figure 3A) and the blood capillary in spleens (Figure 3B), myocardium (Figure 3C), lungs (Figure 3D) and spinal cords (Figure 3E) were dilated, in which massive erythroblasts were accumulated as observed in blood smears. The parenchyma hyperplasia and increased erythroblast were seen in bone marrow (Figure 3F). At high magnification, some of erythroblasts in all tissue sections had mitotic figures.

### Virological assay

**Figure 4:**
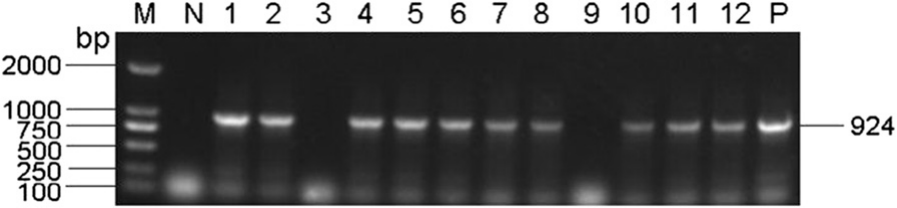
**The result of PCR for ALV-J detection.** M: DL2000 Marker; Lane N: Negative control; Lane P: Positive control; Lane 1–3: two samples from Xintai positive and one negative; Lane 4–6: samples from Sishui all positive; Lane 7–12: five samples from Jinan positive and one negative.

Provirus DNAs extracted from livers of the chickens of all the three flocks were assayed with PCR using AEV, ALV-A, ALV-B, ALV-J, REV and MDV specific primers. The results showed that all the samples tested were negative for AEV, ALV-A, ALV-B, REV and MDV (data not shown). Ten of the twelve samples were positive for ALV-J with a PCR product of 924 bp as expected, one sample from flock 1 and one from flock 2 were PCR negative (Figure 4).

### Antigen distribution and tropism

**Figure 5:**
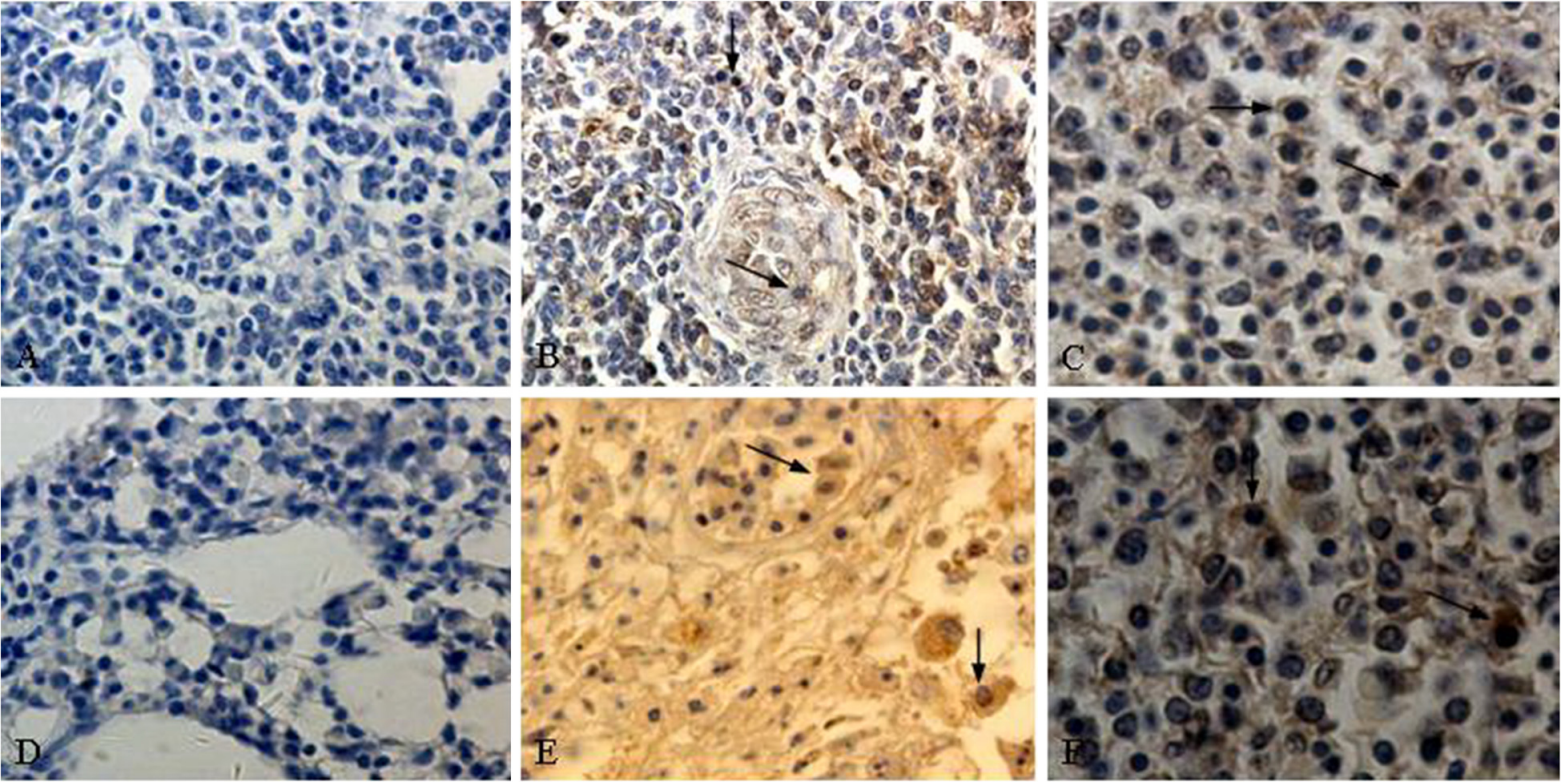
**Immunohistochemistry.** (**A**) Negative control of spleen, IHC, 200×; The cytoplasm and cytomembrane of erythroblast in spleen were positive for ALV-J (**B-C**), IHC, 200× and 400×; (**D**) Negative control of spleen, IHC, 200×; The cytoplasm and cytomembrane of erythroblast in lung were positive for ALV-J (**E-F**), IHC, 200× and 400×.

In order to further detect the distribution of ALV-J antigen in different organs, immunohistochemistry using the anti-ALV-J specific antibody was performed to detect the ALV-J positive signals. The ALV-J positive signals were indicated by the brown staining of the erythroblast cytoplasm in the immunohistochemical stain assays. The results showed that the ALV-J positive signals were mainly presented in spleen (Figure 5B-C), lung (Figure 5E-F) and other tissues especially rich in blood. However, AEV was negative label (data not shown).

## Discussion

The findings in the present paper documented the occurrence of ALV-J-induced erythroblastosis in commercial layer chickens and Chinese local breed chickens. Numerous erythroblasts at different growth stages in the blood, spleen, lung, bone marrow and other organs of the infected birds were observed consistently. Neoplastic lymphocytes were not observed. Thus, Marek’s disease and reticuloendotheliosis were all eliminated through examination of hematology and histology. The PCR results further supported this conclusion.

Several ALV strains have been reported to induce erythroblastosis. These included chronic ALV strains such as RPL12 [12] and RAV-1 [13] that induce erythroblastosis by the activation of cellular oncogene *c-erbB* by LTR insertion [14] and acutely transforming viruses such as AEV-H and AEV-ES4 strains containing *erb-A* and/or *erb-B* oncogenic sequences [15]. To identify the possible viral pathogens of the sick chickens, in the present study we designed primers specific to AEV genes encoding polyproteins gag-p75-erbA and erbB based on the published sequence (Genbank access number: K02006.1) for PCR assays. The negative PCR and immunohistochemistry results of all the tested samples using the AEV specific primers eliminate the infection of AEV in the examined chickens. With the same philosophy and method, we also eliminated the ALV-A and ALV-B infections in the examined chickens. The most PCR reactions of the tested samples (10/12) are positive when using the ALV-J specific primers. Token together, the results of the viral specific PCR assays suggest that the examined chickens were infected with ALV-J.

Disease associated with ALV-J has, since its reported in the last century 90’s [16], become a major problem in chickens worldwide associated with the high oncogenicity and broad carcinoma spectrum. ALV-J predominantly induces a late-onset myelocytomatosis [17] because of their tropism to the cells of the myeloid rather than the lymphoid lineage [2]. During the last 2 years, we have observed, in addition to myelocytomatosis, the occurrence of neoplastic lesions which are the indicative of erythroblastosis in various tissues of chickens from three flocks naturally infected with ALV-J. The results of immunohistochemistry demonstrated that the extension of this tropism of ALV-J strains infected submitted birds for cells of erythroid lineage in vivo. This is the first time that erythroblastosis have been identified as the primary neoplastic lesion induced by ALV-J in China.

Clinically, the erythroblast leukemia is divided into two types: anemia and hyperplasia. The hyperplasia type characterized with the presence of massive erythroblasts in blood was more common than anemia type characterized with rare immature erythrocytes. In this case, significantly increased erythroblasts were observed in histopathological sections of all submitted birds, by which hyperplasia type of erythroblast leukemia was diagnosed.

Venugopal *et al.* have reported that the incidence of erythroblastosis was higher in birds inoculated with the virus after hatching, and it is possible that the transformation of erythroblasts could be dependent on the developmental stage and numbers of the target cells at the time of infection [18]. In this study, the incubation periods of two flocks of Chinese local breed were similar and longer than that of commercial layer chickens. Unfortunately, the time of infection was not clear. The susceptivity of host was maybe an important factor.

## Competing interests

The authors declare that they have no financial or competing interests.

## Authors’ contributions

ZQ Cheng designed the study. GH Wang analysis data and wrote the paper. YP Jiang carried out histopathological examination. LL Yu performed the cell culture and DNA extraction. Y Wang carried out PCR. XM Zhao carried out immunohistochemistry. All the authors have read and approved the final manuscript.
